# Delayed cerebral thrombosis complicating pneumococcal meningitis: an autopsy study

**DOI:** 10.1186/s13613-018-0368-8

**Published:** 2018-02-09

**Authors:** Joo-Yeon Engelen-Lee, Matthijs C. Brouwer, Eleonora Aronica, Diederik van de Beek

**Affiliations:** 1 Department of Neurology, Academic Medical Center, University of Amsterdam, Amsterdam Neuroscience, PO Box 22660, 1100 DD Amsterdam, The Netherlands; 2 Department of Neuropathology, Academic Medical Center, University of Amsterdam, Amsterdam Neuroscience, Amsterdam, The Netherlands; 3 Stichting Epilepsie Instellingen Nederland (SEIN), Cruquius, The Netherlands

**Keywords:** Pneumococcal meningitis, Histopathology, Vascular inflammation

## Abstract

**Background:**

Delayed cerebral thrombosis (DCT) is a devastating cerebrovascular complication in patients with excellent initial recovery of pneumococcal meningitis. The aetiology is unknown, but direct bacterial invasion, activation of coagulation or post-infectious immunoglobulin deposition has been suggested.

**Methods:**

We studied histopathology of 4 patients with pneumococcal meningitis complicated by DCT. Results were compared with 8 patients who died of pneumococcal meningitis without DCT and 3 non-meningitis control cases. Furthermore, we evaluated vascular immunoglobulin depositions (IgA, IgG and IgM) and the presence of pneumococcal capsules by immunofluorescence.

**Results:**

Patients who died after pneumococcal meningitis showed inflammation in the meninges and blood vessels with extensive infarction and thrombosis. We did not observe gross differences between DCT and non-DCT patients, except that 2 of 4 DCT patients had a basilar artery aneurysm compared to none of the non-DCT patients. We observed high density of IgM and IgG deposition in meningitis cases as compared to controls, but no difference between DCT and non-DCT patients. Immunofluorescence staining of pneumococci demonstrated the presence of bacterial capsules in the meninges of all meningitis patients, even 35 days after the initiation of antibiotic treatment.

**Conclusion:**

The aetiology of DCT complicating pneumococcal meningitis seems to be of multifactorial aetiology and includes vascular inflammation, thromboembolism of large arteries and infectious intracranial aneurysms. Pneumococcal cell wall components can be observed for weeks after pneumococcal meningitis and may be a source of resurging inflammation after the initial immunosuppression by dexamethasone.

## Background

Bacterial meningitis is a severe infection of the central nervous system that is most commonly caused by *Streptococcus pneumoniae* (pneumococcus; 70% of cases) [1, 2]. During invasive disease, bacterial nasopharyngeal epithelial adhesion is followed by bloodstream invasion [3]. After crossing the blood–brain barrier (BBB), bacteria multiply freely and trigger activation of immune cells, leading to a massive inflammatory response causing cerebral infarction or seizures and eventually death [4]. In a randomized controlled study, adjunctive anti-inflammatory treatment with dexamethasone was shown to reduce unfavourable outcome with 10% [5, 6]. Implementation of this therapy led to a nationwide decrease in unfavourable outcome of 10% in the Netherlands [7–10]. Nevertheless, case fatality is high (18–25%) and neurological sequelae occur in half of surviving patients [1, 11].

In 2009, we described 6 patients with an excellent initial recovery after pneumococcal meningitis who suddenly deteriorated 7–14 days after admission due to multiple cerebral infarctions [12]. Autopsy in one patient showed an arterial thrombosis in the posterior circulation after which the complication was described as “delayed cerebral thrombosis”. Further research showed that this delayed cerebral thrombosis (DCT) is a rare but devastating complication of bacterial meningitis occurring in 2–4% of patients with pneumococcal meningitis [12, 13]. DCT has been reported in 19 patients of whom 18 had pneumococcal meningitis and adjunctive dexamethasone therapy seems to be a predisposing condition [12, 14]. The theory was postulated that the immunosuppressive effect by dexamethasone during the first 4 days of meningitis treatment wears off after 7–14 days and bacterial fragments cause a resurgence of the inflammatory response. Besides this theory, other aetiologies have been suggested including direct bacterial invasion, activation of coagulation or a post-infectious immunoglobulin deposition [12, 13, 15–18]. To get more insights in the aetiology of this complication, we performed neuropathological examination of brains of four patients with delayed cerebral thrombosis.

## Methods

### Patients

Patients with community-acquired pneumococcal meningitis in whom autopsy was performed between 1985 and 2016 were identified from two nationwide prospective cohort studies and in the neuropathology database of the Academic Medical Center, Amsterdam [5]. The pathology specimens and clinical data of these patients have been collected by MeninGene–PATH Biobank following the methods previously described [19]. Clinical information was studied by two neurologists for case selection of DCT (MCB and DvdB). Next, 8 pneumococcal meningitis patients were selected in whom autopsy was performed after at least 7 days of admission without the typical clinical presentation of initial improvement and deterioration thereafter were selected from the previous study (from here on referred to as non-DCT cases) [19]. For controls of the immunoglobulin staining, non-meningitis control cases without neuropathological abnormalities were chosen from the database of the Department of Neuropathology of the Academic Medical Center, Amsterdam. Histology slides, tissue blocks and autopsy reports were obtained. All the brain autopsies were carried out after receiving informed consent and tissue was obtained and used in accordance with the AMC Research Code and the Declaration of Helsinki.

### Histopathology

The local pathologists examined the brains macroscopically. All brain slices were examined thoroughly with extra attention to areas with known abnormality from clinical and radiological investigation. Standard brain samples were taken according to the local protocol, and extra samples were taken from clinically relevant areas and grossly abnormal areas. Brain tissue samples were formalin-fixed and paraffin-embedded, followed by cutting and haematoxylin–eosin (HE) staining at the local institutes. All available slides and paraffin blocks were collected at the AMC. The availability of sampled brain regions (cortex, hippocampus, basal ganglia, brainstem, spinal cord, sagittal venous sinus, large vessels and meninges) for individual cases was documented. The slides were re-evaluated for meningeal and parenchymal infiltration of inflammatory cells, infarction, haemorrhage, abscess, inflammation of medium–large arteries in the meninges, small parenchymal vessels, thrombosis of arterial, venous and small vascular. These evaluations were scored as described in our previous study [19]. The age and extension of infarction and haemorrhage are documented in the brain areas concerned. For the histological evaluation, a Zeiss Axioskop light microscope with 6 object lenses of magnification of 2×, 4×, 10×, 20×, 40× and 100× and LED light source was used.

### Immunohistology immunoglobulins

Paraffin blocks were cut in 5 µm thickness and mounted on the slides (StarFrost), followed by deparaffinization and rehydration. Blocking of endogenous peroxidase is performed in 0.3% hydrogen peroxide in methanol for 15 min. After antigen retrieval by boiling in 10 mM sodium citrate pH 6 for 10 min, the slides were incubated with 150 µl primary antibodies (rabbit, DAKO, IgA and IgG 1:32,000, IgM 1:16,000) for 60 min. The slides were then incubated for 30 min at room temperature with the ready-for-use PowerVision peroxidise system (HRP, Immunologic), followed by 10 min incubation with chromogen 3.3′-diaminobenzidine (DAB, Sigma), counterstaining with haematoxylin for 10 min and mounting with the coverslip. The slides were scanned with D. Sight fluo scanner/microscope (A. Menarini, Florence, Italy). The same arteries of consecutive slide sections were chosen to compare the amount of deposition of IgA, IgG and IgM immunoglobulins in the arterial layers of tunica intima, media and adventitia.

### Immunofluorescence pneumococcal capsule

Paraffin blocks were cut in 6 µm thickness and mounted on the slides (StarFrost), followed by deparaffinization and rehydration. Blocking of endogenous peroxidase is performed in 0.3% hydrogen peroxide in methanol for 15 min. After antigen retrieval by boiling in 10 mM sodium citrate pH 6 for 10 min, slides were incubated with 150 µl primary antibody (Omni serum, 1:800 diluted, rabbit, Statens Serum Institute, Denmark) for 60 min. The slides were then incubated with 1:400 diluted fluorescent dye (Abcam, anti-rabbit) for 30 min. The slides were washed, air-dried and mounted with the coverslip with a DAPI mounting medium (Vectashield). The slides were visualized with D. Sight fluo scanner/microscope (A. Menarini, Florence, Italy).

## Results

### Case descriptions

Patient 1 was a 39-year-old male who presented with fever, headache, aphasia and right-sided hemiparesis in 2003 (Table 1) [13]. He was treated with penicillin and dexamethasone. Cerebrospinal fluid (CSF) examination was consistent with bacterial meningitis, and CSF culture grew pneumococci, susceptible to penicillin. He showed an excellent clinical recovery with a normal neurological examination. However, at day 14 he developed headache and decreased level of consciousness prompting intubation and ICU admission. Cranial MRI showed multiple areas of cerebral infarction, including the thalami, brain stem and the parietal and occipital lobes. MRA of cerebral arteries shows dilatation of the basilar artery. Supportive care was withdrawn at day 32 after initial admission, after which the patient died.

**Table 1:** Clinical characteristics of four patients with delayed cerebral thrombosis complicating pneumococcal meningitis

Characteristic	Patient 1	Patient 2	Patient 3	Patient 4
Age (years)	39	52	73	68
Gender	Male	Male	Male	Female
Predisposing conditions				
Otitis or sinusitis	Present	Absent	Present	Absent
Immunocompromised	No	Yes	No	No
Glasgow Coma Scale score	11	11	11	13
Neck stiffness	Yes	Yes	Yes	Yes
CSF leucocyte count (cells/mm^3^)	1780	51	17,700	9
Time to secondary deterioration (days)	14	10	7	6
Cranial imaging	MRI: infarction of the thalami, brain stem and cerebral hemispheres MRA: dilatation of the basilar artery	MRI: infarction of the brain stem, cerebellum, basal ganglia and temporal lobes	MRI: infarction in brainstem and cerebral hemispheres	MRI: infarction of the brain stem, thalamus and cerebral hemispheres MRA: convolution of basilar artery
Time to death (days)	32	12	25	16

Patient 2 was a 52-year-old male who presented with fever, headache and altered consciousness in 2006. He was treated with amoxicillin and dexamethasone. CSF examination was consistent with bacterial meningitis, and CSF culture grew pneumococci, susceptible to penicillin. The patient gradually improved over 1 week. On day 10 the patient suddenly deteriorated with a decreased level of consciousness prompting intubation and ICU admission. Cranial MRI showed extensive infarction of the brain stem, cerebellum, basal ganglia and temporal lobes with normal flow in the basilar artery. Supportive care was withdrawn at day 12, and the patient died.

Patient 3 was a 73-year-old male who presented with earache, fever and altered consciousness in 2008 [13]. On admission, he had neck stiffness, global aphasia and gaze deviation to the left. He was treated with penicillin and dexamethasone. CSF examination was consistent with bacterial meningitis, and CSF culture grew pneumococci, susceptible to penicillin. Over the next few days, the patient improved. On day 6, he was awake, alert and ambulating. However, on day 7, fever recurred and he developed gait impairment. On day 8, his level of consciousness started to fluctuate, with intermittent dysconjugate eye movements. On day 12, cranial MRI showed multiple areas of cerebral infarction in brainstem and cerebral hemispheres. Supportive care was withdrawn at day 25, and the patient died.

Patient 4 was a 68-year-old female who presented with a shoulder pain, fever and altered consciousness in 2016. She was treated with ceftriaxone, amoxicillin and dexamethasone. CSF examination showed normal leucocyte count, with low glucose and high protein levels. CSF culture grew pneumococci, susceptible to penicillin. She completely recovered on a week. On day 6, she deteriorated with a decreased level of consciousness. Cranial MRI showed cerebral infarctions in brain stem, thalamus and on several locations in both cerebral hemispheres. MRA taken on the same day demonstrated convolution of basilar artery. Supportive care was withdrawn at day 16, and the patient died.

### Histopathology of cases

We had an average number of 16 brain slides per DCT case, range 17–20 slides. In all cases, cortex, basal ganglia (except patient 3), hippocampus, cerebellum, mesencephalon, pons and medulla oblongata were sampled. In two cases, large cerebral arteries including the basilar artery were sampled. The mean age of the non-DCT meningitis patients was 70 years (range 46–88; 4 women) which was comparable with DCT patients (median 54; range 39–73). The cause of death among non-DCT meningitis patients was known in 7 patients and attributed to systemic complications in 6 patients (86%). Three died due to respiratory failure or pneumonia, and myocardial infarction, ruptured aneurysm of the abdominal aorta and septic shock were the cause of death in 1 patient each. Histopathology of brains of these patients has been described previously [19]. We had an average number of 13 slides of these non-DCT meningitis patients (range 11–15). As non-meningitis controls, 3 cases were selected who all died of ischaemic heart disease.

**Fig. 1:**
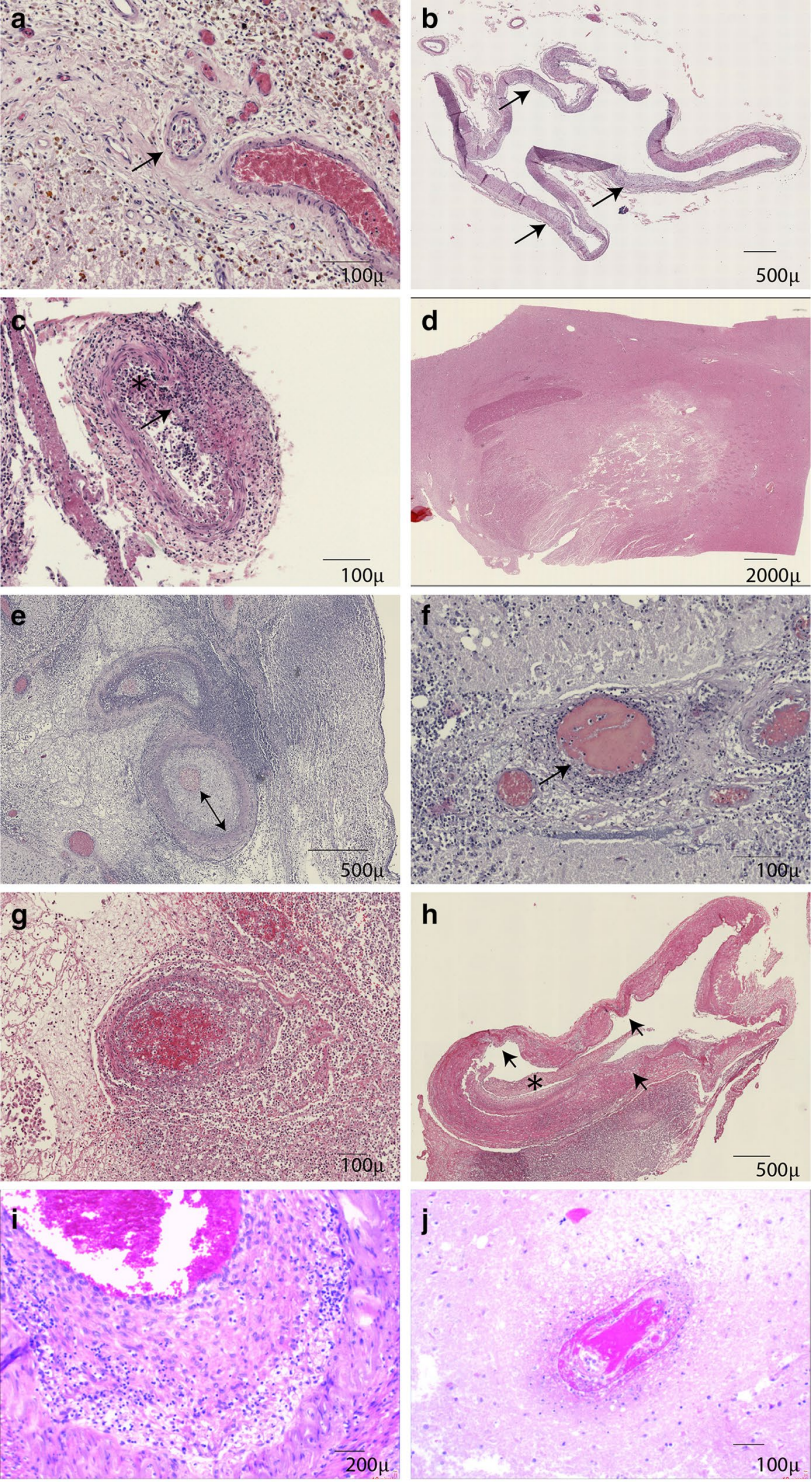
Histology of the four DCT cases. Case 1 (**a** and **b**): **a** small arteries with organized thrombosis are occasionally observed (**a**). **b** The basilar artery showed no active inflammation and was dilated with irregular thickening of endothelial layer (arrow) and disruption of elastic layer. Case 2 (**c** and **d**): **c** the arteries showed still active inflammation with disruption of elastic layer (arrow) and thrombosis (star). **f** Infarction in basal ganglia. Case 3 (**e** and **f**): **e** larger meningeal arteries show often circumferential thickening of intima (arrow) and active inflammation in the media (arrow). **f** Many smaller arteries in the vicinity of the inflamed or destructed arteries show thrombosis with neutrophil infiltrates. Case 4 (**g** and **h**): **g** a severely inflamed artery with vascular destruction and obstruction in the meningeal pus pocket at the frontal lobe showing organization. **h** The basilar artery showed chronic active inflammation and dilatation with disruption of elastic layer (arrows), partially obstructed by thromboembolus (star) with leucocyte clearance. Similarly observed pathologies in non-DCT control meningitis cases (**i** and **j**): **i** active vascular inflammation of a meningeal artery with thickening of intima and inflammation of tunica media. **j** thrombosis of small parenchymal vessel

Histopathology in patient 1 showed minor chronic meningeal inflammation with scanty presence of macrophages, old infarctions in temporal/parietal cortex, basal ganglia, thalamus, mesencephalon, pons, medullar oblongata and cerebellum with clearance by macrophages and no active vascular inflammation (Table 2). Thrombosis with organization was seen, but only in small arteries (Fig. 1a). The basilar artery of this patient showed dilatation with irregular thickening of endothelial layer and disruption of elastic layer without inflammation (Fig. 1b). There were small focal haemorrhages.

**Table 2:** Neuropathological findings of patients with pneumococcal meningitis

Type	Age yrs (M/F)	Days admission-autopsy	DXM	Parenchymal infiltration	Meningeal artery inflammation	Arterial thrombosis	Infarction	Area infarction	Bleeding	Area bleeding	Total pathology score
DCT 1	52, M	10	+	+++	+++	++	+++	Basal ganglia, cerebellum, brainstem	+	Focal minor	19
DCT 2	39, M	35	+	+	++	–	+++	Cortex, basal ganglia, cerebellum, brainstem	+	Focal minor	10
DCT 3	73, M	24	+	+	++	+++	+++	Cortex, cerebellum	+	Focal minor	17
DCT 4	68, F	15	+	+	+++	++	+++	Cortex, basal ganglia, cerebellum, brainstem	+	Focal minor	15
Control	46, M	7	_	++	+++	+++	++	Cortex, basal ganglia, brainstem	+++	Cortex, brainstem, cerebellum	22
Control	78, F	18	+	++	+++	+	+++	Cortex, basal ganglia, cerebellum, brainstem	+++	?	19
Control	73, F	21	+	++	++	++	++	Cortex, basal ganglia, brainstem	+	Brainstem, cerebellum	14
Control	48, F	30	+	++	+++	–	++	Cortex, basal ganglia, cerebellum, brainstem	+++	Basal ganglia	16
Control	88, F	30	?	++	–	++	+	Cortex, cerebellum, brainstem	++	Brainstem, cerebellum	15
Control	75, M	30	?	++	+++	++	+++	Cortex	+	?	19
Control	79, M	12	–	–	+	++	++	Cortex	–	–	14
Control	71, F	15	?	–	+	–	++	Cortex	+	?	6

*DCT* delayed cerebral thrombosis, *DXM* dexamethasone

In patient 2 active inflammation of the meninges was seen with a predominance of neutrophils (Fig. 1c). There were recent infarctions in the basal ganglia (Fig. 1d), brainstem and cerebellum with only focal minor infiltration of neutrophils. The meningeal arteries in the brainstem and cerebellum were severely inflamed with neutrophil infiltration, often with profound vascular destruction (Fig. 1c). Thrombosis in the arteries was observed with vascular inflammation and small focal haemorrhages. A small abscess (diameter 2 mm) in basal ganglia and focal neutrophil infiltration of ventricle were seen.

Patient 3 showed chronic active inflammation in meninges with mixed population of neutrophils and macrophages. There were extensive infarctions in the cortex, basal ganglia, cerebellum, hippocampus and mesencephalon, both recent and of older date. Severe inflammation of larger meningeal arteries with circular thickening of intima (Fig. 1e) was seen with vascular obstruction and destruction and small focal haemorrhages. The smaller arteries showed thrombosis with inflammation (Fig. 1f). Ventricles showed focal infiltration of neutrophils.

Patient 4 showed chronic active inflammation in meninges with the presence of neutrophils mixed with macrophages. There were infarctions present in the frontal lobe, basal ganglia, thalamus, internal capsule, mesencephalon, pons and cerebellum. Meningeal arteries at the sites of meningeal pus pockets in the frontal lobe and cerebellum showed severe inflammation, destruction and obstruction (Fig. 1g). The basilar artery was heavily inflamed with dilatation, irregular thickening of endothelial layer and disruption of elastic layer, infiltrated by neutrophils, macrophages and lymphocytes, and thromboembolism was present with small focal haemorrhages (Fig. 1h). The left medial cerebral artery showed thrombosis and focal mild neutrophil infiltration in intima.

Pathological findings identified in the DCT cases were compared with non-DCT meningitis (Table 2) [12, 13]. All had leucocyte infiltrations, arterial inflammation (except for DCT patient 1), cerebral haemorrhage and infarction. Arterial thrombosis was present in both groups, and extensive infarction in the brainstem, basal ganglia and posterior circulation area were observed in 4 of 4 DCT patients (100%) versus 4 of 8 (50%) of the non-DCT meningitis cases. Two of the 4 DCT patients had basilar artery dilation. None of the non-DCT patients was reported to have basilar artery dilation at original autopsy. Total meningitis pathology scores ranged from 10 to 35 for DCT patients (median, 16) and from 7 to 30 for non-DCT patients (median, 18).

### Immunohistology immunoglobulins

**Fig. 2:**
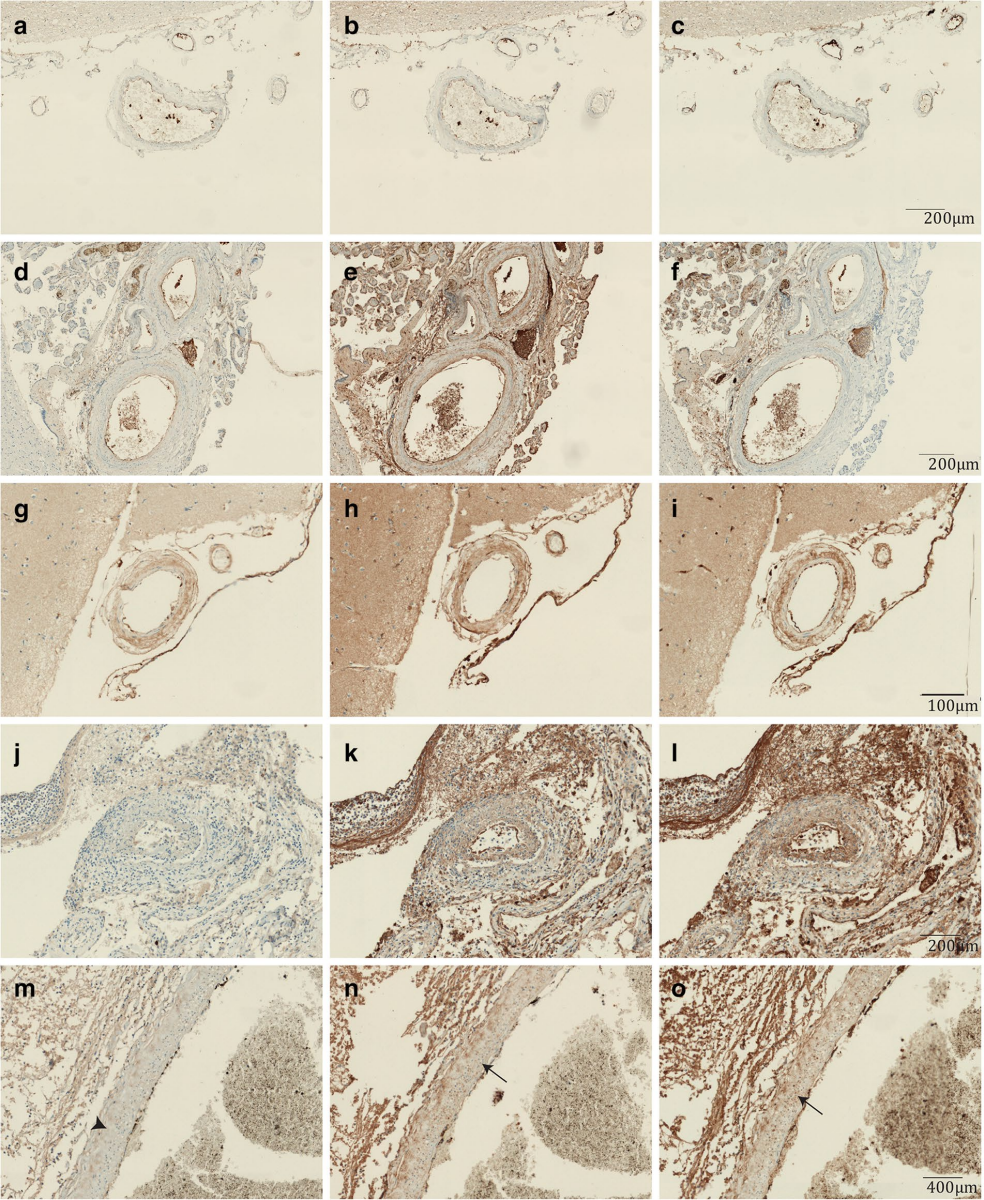
Immunoglobulin staining of control patients, control meningitis patients and DCT patients. **a**–**c** A non-meningitis control case without immunoglobulin deposition. IgA (**a**), IgG (**b**) and IgM (**c**). **d**–**f** A non-meningitis control case with deposition of IgG (**e**). No IgA (**d**) and IgM (**f**) deposition is seen. **g**–**i** A non-meningitis control case with deposition of IgA (**g**), IgG (**h**) and IgM (**i**). IgG is also deposited in tunica media (arrow). **j**–**l** Representative image of immune globulin deposition in both DCT and control meningitis cases. IgG (**k**) and IgM (**l**) deposition can be seen in all three arterial layers. There was stronger IgM stain observed than IgG. IgA (**j**) was in most cases absent. **m**–**o** Immunoglobulin deposition in artery without inflammation. IgG (**n**) and IgM (**o**) were seen in all three arterial layers including media of a non-DCT meningitis case (arrows). Little IgA (**m**) was observed only in tunica adventitia (arrow head)

To explore whether delayed cerebral thrombosis is mediated by immune complex deposition, we performed an IgG, IgM and IgA staining. The non-meningitis control cases showed variable immune complex deposition with no deposition in the first non-meningitis control (Fig. 2a–c), IgG deposition in tunica intima and adventitia in the arteries in a second non-meningitis control case (Fig. 2d–f) and deposition of IgG in all layers and IgM and IgA in the intima and adventitia in the third non-meningitis control (Fig. 2g–i). There was no difference in immunoglobulin deposition patterns between the non-DCT meningitis and DCT cases. All meningitis cases showed deposition of IgM, IgG and IgA, in the tunica intima and adventitia and occasionally also in the media. The highest density was seen with IgM, whereas IgG density was less and IgA was weak or absent in meningitis cases (Fig. 2j–l). The immunoglobulin deposition was present in the arteries with inflammation, but it was also observed in the arteries without inflammation, occasionally in all three arterial layers (Fig. 2m–o).

### Immunofluorescence staining of pneumococcal capsule

**Fig. 3:**
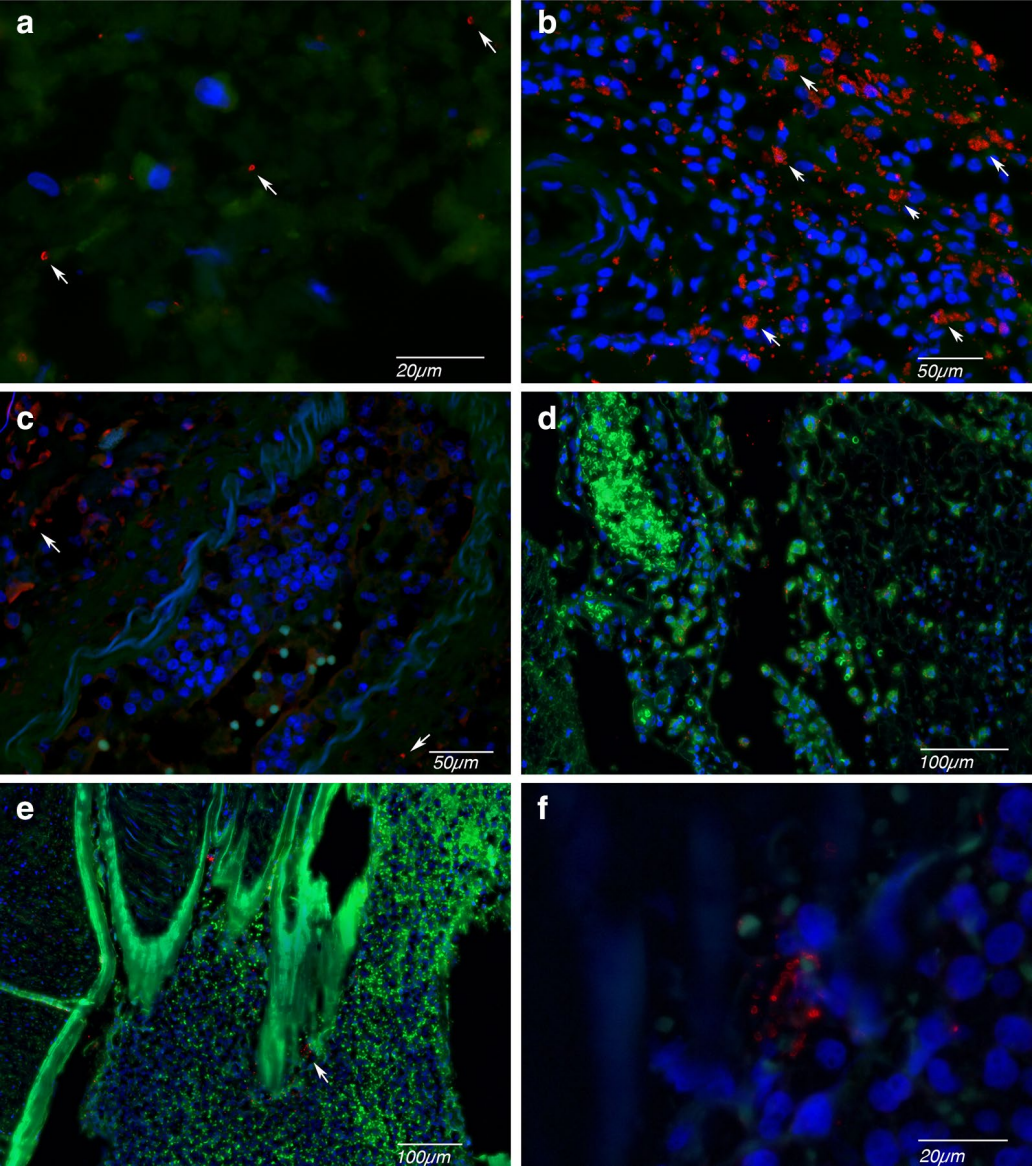
Immunofluorescent analysis pneumococcal capsules of DCT patients and control meningitis patients. **a**–**e** DCT cases with the presence of various amount of intact pneumococcal capsules (a arrow). In case 1 (**a**), pneumococcal capsules were present sporadically. In case 2 (**b**), abundant presence of pneumococcal capsules was observed. In case 3 (**c**) and case 4 (**d**), a moderate amount was seen. In the thromboembolus of the basilar artery of the case 4 (**e**), large groups of bacteria were present (arrow) in addition to isolated bacteria (with mix of macrophages and neutrophils). The group of bacteria was enlarged in inlet. **f** Abundant presence of pneumococcal capsules in a control meningitis case

The presence of pneumococcal capsule was seen in the meninges of all four DCT cases, represented by circular enhancement in immunofluorescent (IF) staining. In the meninges, one patient (patient 2, Fig. 3a) showed abundance of pneumococcal capsules, two patients moderate amount (patient 3 and 4, Fig. 3b, c) and one case sporadic presence of pneumococcal capsule (case 1, Fig. 3d). The pneumococcal capsules were located either isolated or as groups in the cytoplasm of inflammatory cells or extracellularly. The pneumococcal capsules were identified abundantly in the thromboembolism of basilar artery of case 4 (Fig. 3e). A variable amount of pneumococcal capsules was observed in the non-DCT meningitis cases, ranging from abundant (Fig. 3f) to almost none, with the majority of cases demonstrating a moderate amount.

## Discussion

We present four patients with pneumococcal meningitis complicated by DCT. All DCT patients made a good or excellent recovery in the first week of admission and subsequently deteriorated. Histopathology showed arterial inflammation in all patients and multiple cerebral infarcts in cortex, basal ganglia, cerebellum and brain stem.

Half of DCT patients had basilar artery dilatation, an unusual finding in bacterial meningitis that was not observed in the non-DCT cases. The fusiform basilar artery dilatation in the course of bacterial meningitis found in two DCT cases is consistent with the diagnosis of infectious intracranial aneurysm, also known as mycotic aneurysm [20]. Infectious intracranial aneurysms are rare, localized, cerebrovascular lesions that make up 0.7–5.4% of all intracranial aneurysms [21]. The majority of cases (65%) occur in the context of bacterial infectious endocarditis, and meningitis is estimated to be the cause of 5% of infectious intracranial aneurysms [20]. There are few case reports of infectious intracranial aneurysms in pneumococcal meningitis which describe both saccular and fusiform aneurysms [22, 23]. One of our DCT cases showed thrombosis in the basilar aneurysm and its branches which caused the infarction. The origin of the thrombosis cannot be traced, but an embolus or even spontaneous thrombosis of intracranial aneurysm could be suspected [24], especially in the setting of vascular inflammation [25].

Previously published cases describe bleeding from the aneurysm during the course of bacterial meningitis as cause of secondary deterioration, which was not observed in our DCT cases. Our observation stresses the need of imaging using MR angiography to detect infectious aneurysms or more generalized cerebral vasculopathy in patients with bacterial meningitis and clinical deterioration. Described treatment for DCT so far mostly consisted of high-dose steroids to suppress the supposed resurging inflammatory response [12, 13]. Although platelet aggregation inhibitors could be suggested to reduce the risk of thrombosis in all meningitis patients, it is difficult to test whether this treatment is beneficial in such a rare complication. Anticoagulant treatment has previously been associated with increased risk of cerebral haemorrhage in bacterial meningitis and should be avoided [26].

Histopathological findings of delayed cerebral thrombosis resembled arterial inflammation in acute necrotizing vasculitis with fibrinoid necrosis seen in type III hypersensitivity vasculitis [27]. Type III hypersensitivity occurs when there is accumulation of immune complexes (antigen–antibody complexes) that have not been adequately cleared by innate immune cells, giving rise to an inflammatory response and attraction of leucocytes. Indeed, immunoglobulin staining of the meningitis cases showed distinctive increased deposit of IgM and IgG (IgM > IgG) in arteries as compared to the non-meningitis control cases, suggesting some sort of role of antigen-induced vasculitis in pneumococcal meningitis. However, we observed no difference in immunoglobulin deposition patterns between the control meningitis and delayed cerebral thrombosis cases, suggesting that accumulation of immune complexes is not the key driver of delayed cerebral thrombosis.

 We used anti-pneumococcal capsule antibody to demonstrate the presence of pneumococcal cell wall components with circular enhancement in all meningitis cases with considerable differences in density. Groups of intact capsules were present in the cytoplasm of inflammatory cells, and isolated ones seemed to be present extracellularly. We did not observe gross differences in the presence of pneumococcal capsule between patients with and without DCT. In a previous post-mortem study, including 14 patients with pneumococcal meningitis, *S. pneumoniae* has been detected using Gram and silver staining in the subarachnoid, perivascular and ventricular spaces, but not within the brain parenchyma [28]. The presence of pneumococcal capsule in CSF and brain up to 35 days after initiation of antibiotic treatment may be suggested to be a source for an ongoing (or resurging) inflammatory response. The lack of bacterial growth in repeated CSF cultures in DCT patients argues against ongoing bacterial infection [12]. As the pneumococcal capsules were observed in patients with and without DCT, it cannot be the only mechanism leading to DCT. Previously, we described complement factor 5a to be higher in DCT patients and identified genetic variation in the complement factor 5 gene to influence disease course [12, 29]. Potentially, the pneumococcal capsules only elicit a severe secondary inflammatory response in patients genetically prone to be pro-inflammatory.

DCT is a rare but devastating complication of bacterial meningitis. Adjunctive dexamethasone therapy seems to predispose patients with bacterial meningitis to this complication [12]. Infective intracranial aneurysms may be found in DCT patients although the consequences of this finding is unclear as it may be a marker of a more general cerebral vasculopathy or lead to direct infectious thromboembolisms. No evidence for accumulation of immune complexes or bacteria in blood vessels, or for infectious vasculitis, was found as explanation for the secondary deterioration and massive cerebral infarction of these patients. We observed pneumococcal cell wall components up to 35 days after start of antibiotics treatment that may be a source of ongoing or resurging inflammation. Although speculative, our findings indicate a persistent pro-inflammatory reaction that initially is suppressed by adjunctive dexamethasone treatment. Additional immunosuppressive treatment may therefore be beneficial in DCT patients, as previously suggested.

## References

[1] van de Beek D, de Gans J, Spanjaard L, Weisfelt M, Reitsma JB, Vermeulen M (2004). Clinical features and prognostic factors in adults with bacterial meningitis. N Engl J Med.

[2] Bijlsma MW, Brouwer MC, Kasanmoentalib ES, et al. Community-acquired bacterial meningitis in adults in the Netherlands, 2006–2014: a prospective cohort study. The Lancet Infectious diseases 2015. 10.1016/S1473-3099(15)00430-2 26652862

[3] van de Beek D, Brouwer M, Hasbun R, Koedel U, Whitney CG, Wijdicks E (2016). Community-acquired bacterial meningitis. Nat Rev Dis Primers.

[4] Mook-Kanamori BB, Geldhoff M, van der Poll T, van de Beek D (2011). Pathogenesis and pathophysiology of pneumococcal meningitis. Clin Microbiol Rev.

[5] de Gans J, van de Beek D (2002). European dexamethasone in adulthood bacterial meningitis study I. Dexamethasone in adults with bacterial meningitis. N Engl J Med.

[6] van de Beek D, de Gans J, McIntyre P, Prasad K (2004). Steroids in adults with acute bacterial meningitis: a systematic review. Lancet Infect Dis.

[7] Brouwer MC, Heckenberg SG, de Gans J, Spanjaard L, Reitsma JB, van de Beek D (2010). Nationwide implementation of adjunctive dexamethasone therapy for pneumococcal meningitis. Neurology.

[8] van de Beek D, Brouwer MC, Thwaites GE, Tunkel AR (2012). Advances in treatment of bacterial meningitis. Lancet.

[9] Hoogman M, van de Beek D, Weisfelt M, de Gans J, Schmand B (2007). Cognitive outcome in adults after bacterial meningitis. J Neurol Neurosurg Psychiatry.

[10] Lucas MJ, Brouwer MC, van de Beek D (2016). Neurological sequelae of bacterial meningitis. J Infect.

[11] van de Beek D, de Gans J, Tunkel AR, Wijdicks EF (2006). Community-acquired bacterial meningitis in adults. N Engl J Med.

[12] Lucas MJ, Brouwer MC, van de Beek D (2013). Delayed cerebral thrombosis in bacterial meningitis: a prospective cohort study. Intensive Care Med.

[13] Schut ES, Brouwer MC, de Gans J, Florquin S, Troost D, van de Beek D (2009). Delayed cerebral thrombosis after initial good recovery from pneumococcal meningitis. Neurology.

[14] Wittebole X, Duprez T, Hantson P (2016). Delayed cerebral ischaemic injury following apparent recovery from Streptococcus pneumoniae meningitis. Acta Clin Belg.

[15] Steiner I (2009). Past as prologue: delayed stroke as a parainfectious process of bacterial meningitis?. Neurology.

[16] Kawaguchi T, Ogawa Y, Inoue T, Tominaga T (2011). Cerebral arteritis with extremely late onset secondary to bacterial meningitis—case report. Neurol Med Chir (Tokyo).

[17] Rice CM, Ramamoorthi M, Renowden SA, Heywood P, Whone AL, Scolding NJ (2012). Cerebral ischaemia in the context of improving, steroid-treated pneumococcal meningitis. QJM Mon J Assoc Physician.

[18] Kato Y, Takeda H, Dembo T, Tanahashi N (2012). Delayed recurrent ischemic stroke after initial good recovery from pneumococcal meningitis. Intern Med.

[19] Engelen-Lee JY, Brouwer MC, Aronica E, van de Beek D (2016). Pneumococcal meningitis: clinical-pathological correlations (meningene-path). Acta Neuropathol Commun.

[20] Ducruet AF, Hickman ZL, Zacharia BE (2010). Intracranial infectious aneurysms: a comprehensive review. Neurosurg Rev.

[21] Nakahara I, Taha MM, Higashi T (2006). Different modalities of treatment of intracranial mycotic aneurysms: report of 4 cases. Surg Neurol.

[22] Palacios A, Llorente AM, Ordonez O, Martinez de Aragon A (2017). Intracranial mycotic aneurysm in a 5 month-old infant with pneumococcal meningitis. Enferm Infecc Microbiol Clin.

[23] Choi KH, Park MS, Kim JT (2012). Infectious intracranial aneurysm presenting with a series of strokes as a complication of pneumococcal meningitis. Eur Neurol.

[24] Cohen JE, Itshayek E, Gomori JM (2007). Spontaneous thrombosis of cerebral aneurysms presenting with ischemic stroke. J Neurol Sci.

[25] Emmi G, Silvestri E, Squatrito D (2015). Thrombosis in vasculitis: from pathogenesis to treatment. Thromb J.

[26] Mook-Kanamori BB, Fritz D, Brouwer MC, van der Ende A, van de Beek D (2012). Intracerebral hemorrhages in adults with community associated bacterial meningitis in adults: should we reconsider anticoagulant therapy?. PLoS One.

[27] Kumar V, Aster JC (2014). Robbins & Cotran. Pathologic basis of disease.

[28] Guarner J, Liu L, Bhatnagar J (2013). Neutrophilic bacterial meningitis: pathology and etiologic diagnosis of fatal cases. Mod Pathol.

[29] Woehrl B, Brouwer MC, Murr C (2011). Complement component 5 contributes to poor disease outcome in humans and mice with pneumococcal meningitis. J Clin Invest.

